# Knotting the MECO Network

**DOI:** 10.3390/e23020141

**Published:** 2021-01-24

**Authors:** Reinhard Folk

**Affiliations:** Institute for Theoretical Physics, Johannes Kepler University Linz, 4040 Linz, Austria; r.folk@liwest.at

**Keywords:** methods and models of statistical physics, interdisciplinary applications of statistical physics, emergence, scaling, complex systems

## Abstract

The Conferences of the Middle European Cooperation in Statistical Physics (MECO) were created as an attempt to establish and maintain an exchange between scientists in the fields of statistical and condensed matter physics from Western and Eastern countries, overcoming the hurdles of the Iron Curtain. Based on personal remembrance and historical resources, the genesis and further development of MECO meetings is described. The annual meetings were interrupted in 1991 by the Yugoslav War but were re-established in 1993 and continue today. Although the fall of the Iron Curtain and the European Research programs changed the situation for the meetings considerably, the ties created by MECO still are useful to help scientific exchange. The history of European (and not only) statistical physics and the history of the MECO are tightly intertwined. It started in a time where an essential breakthrough has been achieved in statistical physics describing the features near phase transitions. In addition to the merging of solid-state physics and field theory concepts, the application of numerical methods (Monte Carlo methods) added a new pillar besides exact solutions and experiments to check theoretical models. In the following, the scientific emphasis (in general) has changed from the traditional fields of the first MECO to complexity and interdisciplinary themes as well.

## 1. Introduction

“During my lectures when I am describing a physical phenomenon, discovery or technological invention I always try to connect with the face(s) of the person(s) who made major contributions in that field and give a little bit of its history.” (Hattice Altug)

There are, according to the historian Lynn Nyhart, three leading historiographic themes in the history of science: how scientific knowledge has been made (and how knowledge has been made scientific), how it has been moved, and how making and moving scientific knowledge have been understood together [1]. Science may be considered as a complex system [2]. In order to study such systems in history, new techniques coming from physics are applied. MECO speakers like Eugene Stanley, coauthor of Science of Science [3], or Albert-László Barabasi, coauthor of the Census of Physics [4], have contributed to this new analysis of history of science. Extending the classical work Little Science, Big Science of Derek de Solla Price [5], these modern techniques of data analysis are applied to historical grown networks of scientists and their publications [6] in order to quantify and predict the dynamics of scientific research.

Statistical methods help to find more general insight into the development of a scientific field and its extension to other fields created by fate and the career of individual scientists in a changing surrounding. The formation and dynamics of networks are of fundamental importance for science. On a large scale they have also been considered as Epistemic Web for production, circulation, and accumulation of knowledge [7].

Different networks are interwoven (e.g., the directed network created by the genealogic network defined by the supervisor-graduate student relation, the open network created by periodic conferences, the collaboration network, the citation network, etc.). Their function is based on the entanglement and interaction of these networks. All these networks are embedded in a political, cultural, and social surrounding hindering or supporting their developments. In addition the technical progress of communication media (e.g., from letters to email) is an accelerating force in the networking processes.

Conferences and/or schools play an important role in sharing knowledge. Most important is the exchange and discussion of preliminary results of research prior to publication. One also should have this in mind in particular for time periods before communication via internet or dissemination of publications on e-Print servers was possible.

The network of the Middle European Collaboration in Statistical Physics (MECO) is in fact one small part of the global scientific network. It was created bottom-up by scientists for strengthening research and collaboration of certain MECO centers’ on both sides of the Iron Curtain in a well-defined field of condensed matter physics. In this paper, based on personal memories of the author as well as other documents, the origin and development of the conference is described. This paper is embedded in a larger project [8], which also uses scientometric methods to analyze the networking.

## 2. Sharing Science in the Time of the Cold War

### 2.1. The *Triangle Seminar* a Model for Supranational Structures in Europe

**Figure 1:**
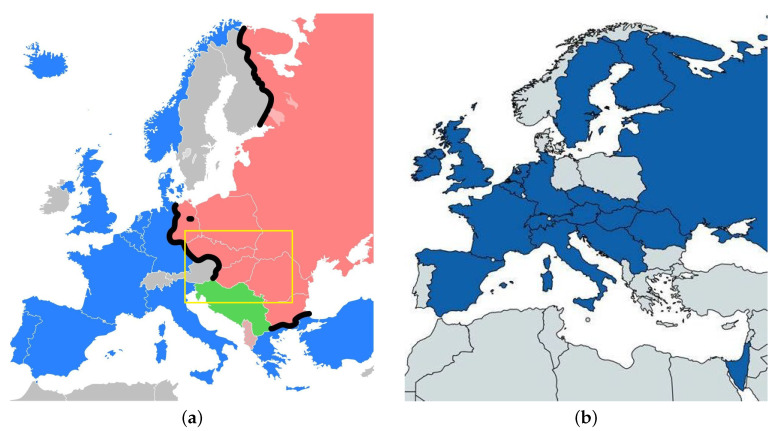
(**a**) Map of the Iron Curtain separating Western European countries (blue: NATO members; grey: Neutral countries) from Eastern European countries (red: Warsaw Pact countries, Yugoslavia; light red: Albania); the yellow frame indicates the region of the academic network of the Habsburg Empire. (**b**) Map of the 18 countries (blue) whose representatives signed the constitution of the European Physical Society on 26 September 1968 from [9]. The map was produced by historicalmapchart.net (https://historicalmapchart.net/world-cold-war.html).

Europe after the Second World War was separated in two parts (the Eastern and Western) by the so called Iron Curtain. Although there is no general definition of the two parts of Europe, the Eastern part is here defined by the communist countries of the Warsaw Pact and Yugoslavia, whereas the Western European countries are all the European countries west of them (including Greece). The Iron Curtain also cut off the academic network that existed within the extension of the Habsburg Empire 1910 before the world wars (see Figure 1a the region within the yellow frame). Of course this old network was strongly connected to the other European countries at that time.

Soon after the war, attempts were made to re-establish such connections, at least in science. “As early as the mid-1950s, the Rockefeller Foundation suggested Austrian, to set up a supranational scientific organization, which should prepare the experience from the history of the Habsburg monarchy for emergence and decay, performance and failure of a multinational empire, for planning and acting in the present and future. The history of the Habsburg monarchy should serve as ‘model case’ for the functioning of supranational structures in Europe.” And indeed, “Soon after the signing of the Austrian State Treaty the first semi-official contacts began in May 1955 from Austrian scientists to colleagues in Czechoslovakia, Hungary, Yugoslavia and Poland. The Minister of Education—responsible for the science agendas—Dr. Heinrich Drimmel supported this establishing of more contacts, so to speak ‘through the Iron Curtain’” ([10] p. 542 translation by the author).

The University of Vienna and here the Physics Department played an important role in the academic landscape of the Habsburg Empire. Thus, it is comprehensible that it could take over the initiative for such connection between Budapest, Bratislava, and Vienna. J. Šebesta informed about this collaboration project at the 2nd International Conference of the European Society for the History of Science [11,12]: “First step[s] to establishing contacts [through the Iron Curtain] were done in 1964. One of the pioneers of this collaboration Prof. Walter Thirring (Institute of Physics, Vienna University) informed me that his father Prof. Hans Thirring at that time disposed with money from Ford foundation and he used it for financial supporting of contacts with East European states …The first lecture which might be called ‘triangle’ took place in June 1968 in Bratislava.”

The holding of this seminar was positively commented on: “During the ‘Prague Spring’ in the second half of 1960s, contacts developed with physicists from western countries, leading to a fruitful triangular collaboration between Vienna, Budapest and Bratislava and regular meetings between top physicists from East and West. In 1968, discussions of a possible Czechoslovak membership of CERN were interrupted by the dramatic political changes in the country” [13], and “The scientific value of this collaboration is highly rated and it has been recommended by UNESCO (and others) as a model for regional scientific cooperation” [14].

Without interruption the Triangle Seminar took place annually between 1968 and 2003. Then it was followed by the Central European Seminar, which is intended to provide stimulating interactions between leading researchers and promising junior physicists.

### 2.2. The *United Nations Paper*

**Figure 2:**
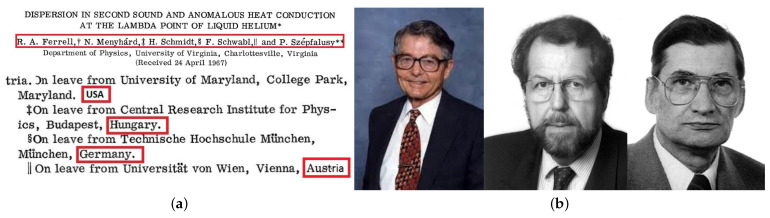
(**a**) The United Nations Paper and (**b**) three of its authors: Richard Ferrell, Franz Schwabl, and Peter Szépfalusy.

There are further prominent exceptions of scientific contacts through the Iron Curtain depending on the eastern country, the academic institutions, and the person, e.g., for Hungarian scientists at that time, Hungarian rules did allow a researcher to spend two years abroad, but not more [15]. Such an example is documented in the so-called United Nations Paper [16] (see Figure 2). Kurt Binder noted [17]: “What is perhaps most remarkable about this paper, it is a rare example of a successful collaboration across the Iron curtain at the climax of the cold war (R.A. Ferrell is from the U.S., but N. Menyhard and P. Szépfalusy are from Hungary, [F. Schwabl from Austria and H. Schmidt from Germany]), roughly at the time when Soviet tanks invaded the Czech republic to extinguish what was called the ‘Spring of Prague’.” Another example of an Eastern Europe scientist is given by the stay of Sava Milošević (from Belgrade (YU) stayed 1969–1971 at MIT (Boston, MA, USA) where he got his Physics degree. The supervisor was H. E. Stanley.)

The topic treated in the United Nations Paper [18]—critical dynamics near the superfluid transition—led to immediate further developments by Halperin and Hohenberg; they wrote, “ In this Letter we outline a general theory of dynamical properties, which seems to us to be the simplest generalization of the static scaling laws, and whose application to the lambda transition reproduces the essential predictions of Ferrell et al.” [19]. How far the scientific content of this paper reached may be demonstrated by W. H. Zurek’s letter in the year 1985 on ‘Cosmological experiments in superfluid helium’ [20] and his contribution at MECO 43 ‘Universality of phase transition dynamics: topological defects from symmetry breaking’.

After the European coauthors returned back to Europe, the collaboration between them was maintained as will be seen below. They remained working in statistical physics and critical phenomena. For example, common papers were published by F. Schwabl together with P. Szépfalusy or together with H. Schmidt.

### 2.3. Schools and Conferences in Western Europe

Despite social and political unrest across the globe, on 26 September 1968, sixty-two physicists gathered in Geneva to found the European Physical Society (EPS) [9]. Among these were the official representatives of the national physical societies of eighteen countries of both Eastern and Western Europe.

At that time, it was one of the first international scientific institutions that was specifically European and at the same time transcended the Cold War political divide. It was founded at a particularly dramatic historical moment: only one month after the armed invasion of Czechoslovakia by five countries of the Warsaw Pact (East Germany, Bulgaria, Hungary, Poland, and USSR). After some discussion it was decided to ‘unify all the countries on basis of physics’, and finally the countries shown in Figure 1b participated. In the following years also institutions of the remaining countries joined the EPS.

The first Europhysics Conference was held in Florence on 14–17 September 1971 by the Condensed Matter Division [21]. “600 delegates some eight plenary and 24 sectional invited papers were presented in addition to about 170 contributed papers.” One topic was Phase Transitions presented by H. Thomas later speaker (MECO 2) and one of the first advisory board members of MECO. A resume of this meeting was “A three-year interval for big meetings was felt to be reasonable, although at the outset a shorter period might be justified; also a balance between East and West was needed.”

In response to this experience a scheme of four categories of conferences was established by the council of the EPS: Divisional, Topical, Study and Summer School [22,23]. With the MECO meetings the so-called Europhysics Study Conferences (previously known as Europhysics Conferences) are most comparable. “Their distinguishing feature is that the formal presentations are specifically designed to stimulate discussion… To make the study group concept workable, the subjects for Europhysics Study Conferences must be specific and well-defined and the attendance (by invitation only) should be restricted to about 70 [up to 100] active workers in the field under discussion… The number of participants from any single country should not exceed one-third of the total …There should be no published proceedings of Europhysics Study Conferences, unless the participants specifically decide to do so.”

#### 2.3.1. The Battelle Geneve Colloquium on Critical Phenomena, 1970

On 12 September 1970 van Hove closed the Battelle Colloquium in Geneva and Gstaad on Critical Phenomena with an after dinner address under the title ‘The Changing Face of Physics’ [24]. One may also refer to Price [5] when one wants to described the changes in the face of physics in the decades before 1970. Similarly, such a title could have been chosen for the story of MECO when it came to ‘her face’ after its inception in 1974. The topics treated at Battelle Colloquium were close to the topics of MECO since it was organized in honor of Lars Onsager. It was concerned with static and dynamic critical phenomena in alloys, magnets, and superconductors. For van Hove the situation concerning critical phenomena looked more like ‘chemistry’ since no fundamental principle was visible. However, a glimpse of the future developments could have been seen in the contribution of G. Jona-Lasinio with the title ‘Renormalization group and theory of phase transitions’ [25]. Some order in the different phenomena was brought in by the talk of H. E. Stanley when explaining ‘universality’. R. A. Ferrell was chairman of the agenda discussion on magnets and superconductors. No eastern scientist is named under the 59 participants.

Elliott Montroll gave at the end of the school a summary where he speculated ([26] p. 646): “There might be three little clouds on the horizon, however. One has to do with the three-dimensional Ising model…A second has to do with the relations between laws of force, lattice structure, and critical exponents…The third has to do with nonlinear processes, …dynamical scaling [is such a problem].” Indeed all these clouds floated in the sky still when MECO started its work.

#### 2.3.2. The Varrenna Schools, 1970 and 1973

**Figure 3:**
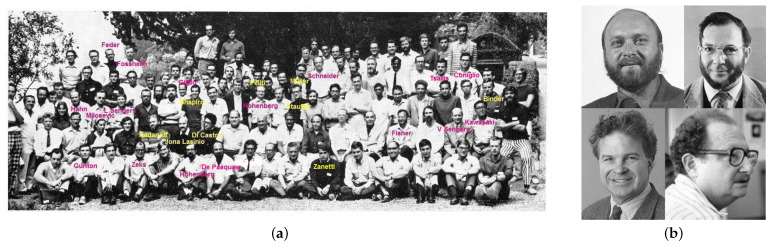
(**a**) Participants of the Varenna School, 1970 ©Italien Physical Society; (**b**) speakers Kadanoff, Fisher, Stanley, and Hohenberg.

One month earlier than the Battelle Colloquium a very important contact for ‘moving and sharing knowledge’ [27] was the Varenna School on Critical Phenomena in 1970 [28]. The most recent advances in the theory of phase transitions, the so-called critical phenomena, were presented by Kadanoff, Stanley, Fisher, Hohenberg, Jona-Lasino, and Di Castro (see Figure 3).

Domb [29,30] noted in his historical review on ‘Critical Phenomena’: “A suggestion that RG [renormalization group] could be relevant to critical phenomena was made at the summer school which Mel Green organized in 1970 in Varenna (de Pasquale, di Castro and Jona-Lasinio 1971) [[31] citation by the author]. However, no precise indication was forthcoming as to how it should be used.” Binder [17] remembered Jona-Lasinio’s lecture: “…although some lectures on mathematical aspects of the renormalization group were given by Giovanni Jona-Lasinio, it remained obscure—at least for me—how this ever could help to understand critical phenomena. Only a year later the papers by Kenneth G. Wilson provided a breakthrough in understanding, and thus the Proceedings Volume of this famous summer school unfortunately became outdated already when it appeared in print (1971).”

Indeed, also Di Castro remembers: “It was rather cold. It may have been our fault. Perhaps we should have been more detailed and specific in establishing the connection between field theory and statistical mechanics, not giving it for granted. We didn’t consider the fact that condensed matter and statistical mechanics physicists were not ready for our ideas” [32]. See also Jona-Lasinio’s answer in the discussion section to his Battelle lecture [25]. Wilson wrote in his Nobel lecture [33]: “In the fall of 1970 Ben Widom [he gave the rapporteur’s introduction ‘Thermodynamics and Scaling’ at the Battelle colloquium [34] and had heard Jona-Lasino’s lecture there] asked me to address his statistical mechanics seminar on the renormalization group. He was particularly interested because Di Castro and Jona-Lasinio had proposed applying the field theoretic renormalization group formalism to critical phenomena, but no one in Widom’s group could understand Di Castro and Jona-Lasinio’s paper.”

Later there was a debate if “Wilson’s final breakthrough was somehow anticipated by Di Castro and Jona-Lasinio” (see Fisher’s review [35]) By all means A. Z. Patashinski (who together with V. L. Pokrovski made important improvements before Wilson) judged: “For the field theory, I would say the formulation of DiCastro and Jona-Lasinio is perfect. They had not calculated exponents, but the scheme is a closed one” [36].

Several speakers and/or participants of this Varenna school (see Figure 3) have been speakers, participants, and/or organizers of the MECO meetings, among them K.A. Müller, G. Jona-Lasinio, C. Di Castro, S. M. Shapiro, S. Milošević, and K. Binder (all MECO 1), R. Kind (MECO 2), D. Stauffer (MECO 3), B. Žekš, M. Giglio ( all MECO 6), L. Peliti, M. Zanetti, F. De Pasquale, A. Coniglio (all MECO 7), K. Fossheim (MEC0 8), J. D. Gunton (MECO 9), M.E. Fisher (MECO 18), J.V. Sengers (MECO 20), C. Tsallis (MECO 25). K. Binder as a young scientist had the opportunity to present the results of his thesis (approved at the Technical University Vienna 1969) at the school. This led 1971 during a stay of P.C. Hohenberg as Visiting Professor in Munich to a fruitful cooperation [17]. This is an example of the importance for young scientists of the possibility to present their results at such a meeting to already established scientists. However from the list of speakers and participants one sees that, apart from Milošević (and he was in fact in the USA), none of the Eastern countries could take part.

In 1973, further sharing of knowledge in Varenna took place [37]. R. O. Davies [38] reviewed this School as follows: “The central theme of this fine collection of papers is the contribution of ‘nondiffraction’ techniques to the study of phase transitions: nuclear magnetic resonance, paramagnetic resonance and birefringence. Most of the emphasis is—very naturally—placed on magnetic and ferroelectric transitions, but there are some articles on liquid crystals and one after-dinner speech on cooperative phenomena in biology.” This after-dinner speech was given by H. E. Stanley and ended with the remark: “In summary, one useful contribution I believe that physicists can make to understanding to mysteries of biology is to focus attention on questions that can be answered with hitherto untried experimental techniques. …as the field of biological physics emerges and appropriate synergisms are created between workers from different backgrounds, I imagine that progress will follow in the same fashion as occurred in the field of critical phenomena [39].” One may point to MECO 44 in the year 2019 to verify this foresight of the interdisciplinary development of the field of phase transitions.

Contrary to the 1970 Varenna school, this time more talks on experimental results were presented. Again, a lot of MECO founders and participants were present: K. A. Müller, R. Blinc, P. Frankus (all MECO 1), K. H. Höck, K. H. Michel, R. Kind, H. Thomas, E. Courtens (all MECO 2), A. Rigamonti (MECO 3) I. Kondor, L. Sasvari, W. Windsch (all MECO 4). J. M. Kosterlitz (MECO 6), H. E. Stanley (MECO 17), M. Coldea (from Cluj-Napóca the host city of MECO 45). Compared to the school in 1970 there were a handful more participants of Eastern countries, namely from Yugoslavia, Hungary, Romania, and Eastern Germany.

#### 2.3.3. The NATO Advanced Study Institute at Geilo, 1971 and 1973

Besides the ‘old’ Varenna School (it was founded 1953) the NATO Advanced Institute organized a series of Schools devoted to phase transitions. The first was held in Geilo 1971 [40]. Its topic ‘Structural Phase Transitions and Soft Modes’ was described at the sixth Geilo school from this perspective ten years later: “…at the first Geilo school, the report of a central peak in the fluctuation spectrum of SrTiO${}_{3}$ close to its 106 K structural phase transition demonstrated that the simple soft-mode theory of such transitions was incomplete. The missing ingredient was the essential nonlinearity of the system” [41]. This *central peak* was for the next years a hot topic.

Among the participants/speakers of the school one finds several scientists that joined the MECO meetings, either as organizers, speakers, or participants: R. Blinc, K. A. Müller, M. W. Valenta (all MECO 1), H. Thomas, N. Szabo (all MECO 2), B. Lavrenčič (MECO 4), R. A. Cowley (MECO 8), T. Schneider, T. Riste (all MECO 9). Only three participants of 63 were from Eastern countries: two from Yugoslavia (R. Blinc, B. Lavrenčič) and one from Poland (A. Wanic).

A follow-up to of the 1971 NATO School was organized 1973 with the topic ‘Anharmonic Lattices, Structural Transitions and Melting’ [42] just the year when the author finished his physics degree studies at the University of Vienna in Theoretical Physics and got a position at the Johannes Kepler University in Linz in the group of his supervisor F. Schwabl. After his postdoctoral stay in the States, Schwabl returned to the University of Vienna for habilitation. Then in 1972 he worked in the group of H. Wagner at the Jülich Research Center. A year later he got the chair of Theoretical Physics at the University in Linz. Some of the research topics there were structural phase transitions and, especially, the central peak problem.

Among the participants/speakers of the School, again one finds K. A. Müller, F. Schwabl, S. M. Shapiro, M. W. Valenta, R. Folk (MECO 1), Fossheim (MECO 8) Hüller (MECO 2) Michel (MECO 2), B. Dorner (MECO 4), R. Kragler (MECO 2), T. Riste (MECO 9), K. Fossheim (MECO 8), A. Hüller (MECO 2), K. H. Michel (MECO 2). None of the 71 participants was from an Eastern country.

The ‘central peak’ was still the hot topic at this second NATO school. “Recent neutron scattering measurements of the soft phonon mode dynamics have revealed a very narrow divergent ‘quasielastic’ component in addition to the expected collapsing phonon sidebands. The phenomenon is only superficially similar to the familiar Rayleigh component of critical scattering in fluids, since in all cases observed so far in solids there is no linear coupling between energy and order parameter fluctuations” [43].

The status of experimental and theoretical results on this critical component in several materials was the theme of the school. As the author began working with Schwabl on this problem, having the opportunity to attend this school was of great help.

#### 2.3.4. The Van der Waals Centennial Conference and the Third International Meeting on Ferroelectricity, 1973

“When it became clear that Professor Voronel was unlikely to be allowed to attend this Conference in person to present his paper the suggestion was made that the paper should be read for him.” C. Domb read the paper at the Van der Waals Centennial Conference on Statistical Mechanics in Amsterdam 27–31 August 1973 [44]. This remark in the proceedings of the conference shows that the situation in Europe was far from free exchange for scientists even when they where invited. This IUPAP (International Union of Pure and Applied Physics) conference had 386 participants; 16 were invited and 83 contributed talks.

IUPAP was established in 1922 in Brussels with 13 Member countries including only Poland from Eastern Europe. Other eastern countries had also joined: Romania 1947, Hungary 1948, Yugoslavia 1954, Soviet Union 1957, and East Germany 1960.

Voronel’s talk was the only one of an eastern country under the 16 invited. Voronel found 1962 the divergence of the specific heat of argon at T${}_{c}$. However, his results were an important step in the experimental confirmation of the understanding of critical phenomena. Under the 83 contributed talks, only three contributions from eastern countries were mentioned: one from Poland by J. Steki and B. Malesinska, one from Yugoslavia by S. Milošević, and one from Hungary by P. Szépfalusy and I. Kondor. This showed that it was still necessary to strengthen the opportunities for Eastern scientists to participate in the exchange of knowledge at conferences organized in Western countries.

**Figure 4:**
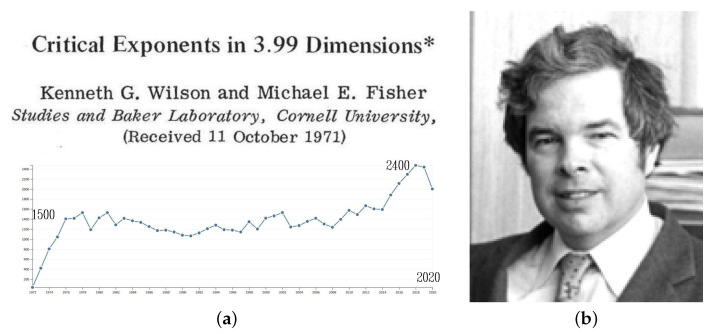
(**a**) Publication on the calculation of critical indices in [47] (the title is attributed to Wilson [48]) below the citations of this paper up to now and (**b**) Kenneth G. Wilson.

K. G. Wilson gave the talk ‘Critical Phenomena in 3.99 Dimensions’ where he explained in more detail how renormalization theory could overcome the breakdown of Landau theory. Apart from inducing a flood of calculations of critical exponents in various systems, Wilson’s theory not only “gave new life to the theory of critical phenomena” [45] but led also advance in other fields like high-energy physics or physics of turbulence. This wide-reaching aspect is reflected in the citation rate (see Figure 4a) and recently in Paul Fendley’s judgment: “The RG is not only the way to understand critical phenomena quantitatively, but lies at the very core of why theoretical physics is so effective” [46].

Another big conference in the same year was the Third International Meeting on Ferroelectricity—an EPS conference—where the following speakers and/or participants could be found: P. Frankus, F. Schwabl, M. W. Valenta (A), R. Blinc (Yu), K. A. Müller, M. Shapiro (USA) MECO 1], J. Fousek [MECO 4], V. Dvorak [MECO 4] (CZ), I. Zheludev [Meco 3], L. Shuvalov (USSR); K. Lukaszewicz [MECO 5] (Poland); W. Rehwald [MECO 2] (CH), E. F. Steigmeier [MECO 4]; D. Bäuerle [MECO 9] (West Germany); W. Windsch [MECO 11] (East Germany); J. C. Toledano, R. Pic, J. Villain [MECO 6] (F).

The International as well as the European Ferroelectricity Meetings (IMF respectively EMF) always had a large number of participants from countries behind the Iron Curtain, as can be seen from lists presented the historical project on the EMFs and IMFs [49]. Jan Fousek (one of the organizers of MECO 13) remembers the First IMF in Prag 1966, which was organized together with V. Dvorák (later advisory board member of MECO): “It was probably this very meeting which opened - even though in a limited extent—the door for establishing contacts between Westeuropean and American scientists on one side and those from ‘behind the Iron curtain’ on the other.” This conference was followed by the first EMF 1969 in Saarbrücken. In the author index of the speakers one finds V. Dvorák, J. Fousek, K. A. Müller, A. Rigamonti, H. Thomas, H. G. Unruh, J. Villain, and W. Windsch.

However, one may conclude from these examples in the field of critical phenomena that there was a lack of possibilities for aspiring scientists to get informed on recent successes in their fields of research by the leading researchers face to face. This holds especially for those from Eastern countries.

### 2.4. The Zeroth MECO in Budapest, 1973

“After his [Szépfalusy’s] return [from USA] to Hungary his activity was centered around the dynamical renormalization group. …In the years when the end of the cold war belonged to the realm of phantasy, Peter Szépfalusy strove to establish and maintain international scientific contacts. He played an instrumental role in launching the Middle European Cooperation in Statistical Physics (MECO) …” [50]. These contacts also included F. Schwabl, who had returned to the University in Vienna at that time. Both were of course interested in the recent developments in the understanding the physics of phase transitions. The most important task was to achieve a form of meeting also open for young scientist of the eastern part of Europe like the Triangle Seminar.

**Figure 5:**
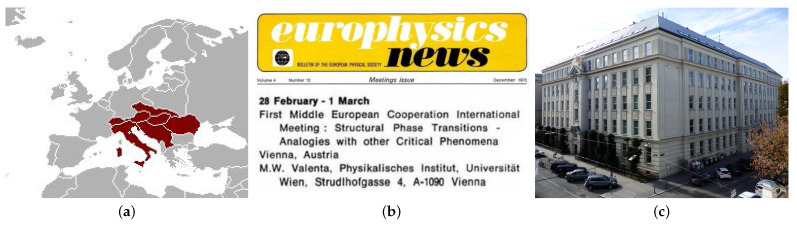
(**a**) The countries of the Mitropa Cup in the years 1927–1940 were considered for the definition of ‘Middle European Countries’ by the founders of MECO. (**b**) The announcement in *europhysics news* [52] and (**c**) the place of the first MECO at the Physics Institute of the University of Vienna (©Dr. Bernd Gross).

Zoltan Rasz reminds “It was Peter Szépfalusy who had the idea that we should organize a meeting with the ‘critical phenomena people’ of the neighboring (Middle-European) countries. The first questions to be settled was what was the Middle of Europe. After some discussion, we decided that there was the Middle European Cup in soccer, and we shall consider those countries which participate in this Cup (It was conducted among the successor states of the former Austria-Hungary! See Figure 5a). The communication was rather slow at that time and random to some extent. e.g., I was attending a Summer School in Varenna in August of 1972 (History of Science) organized partly by Jona-Lasinio. Peter Szépfalusy told me to ask him to visit us. Jona-Lasinio declined but he suggested two of his talented PhD students and, indeed, Attilio L. Stella and G. Benettin came to the 0th MECO meeting.” Jona Lasinio, Gallavotti, and these students just published a paper which proved that the evaluation of the magnetization of the 2d Ising model via the correlations agrees with the one calculated via the free energy (as Ernst Ising in his thesis tried) [51]. Unfortunately, it is not known who from Vienna was present at this meeting (it might have been Valenta) since at the end of 1973 the conference was announced in *europhysics news* (see Figure 5b).

## 3. The First MECO in Vienna, 1974

One may characterize the first MECO as an amalgamation of scientists from the International Meetings on Ferroelectricity, the NATO Schools, and the Varenna Schools. It brought together working groups in experimental physics, in theoretical (or mathematical) physics, and scientists working with simulation methods on computers to achieve the ‘crossfertilization’ as Domb (see [29] p. 50) called it. An example may be as follows: The critical exponent of the order parameter can be measured, calculated analytically (if not exact then approximately) or calculated by analyzing computer simulations. The comparison of the analytic result with experiments and computer results tests the validity of the underlying theoretical model. Such an approach gave rise to new research programs in all the three disciplines and is precisely reflected in the program of the first MECO.

However, in the last years the weight between these three directions changed evidently. This is connected to the individual academic career of the participating scientists and to the possible growth of the ‘physical tree’. In ‘Taking census of physics’ [4] the authors said “We find that the majority of physicists began their careers in only three subfields, branching out to other areas at later career stages, with different rates and transition times.” MECO meetings seem to be an ideal example to study such aspects. MECO accompanied many careers for a long time starting on a branch of the physical tree and extended the branches. Following the limbs for two leaves on the tree connected at present might uncover connections in the past (see below). Apart from that, it contributed to the network knotting in science. However it started from established connections (see the following chapter) in a narrow but strongly expanding field. Thus, MECO is a perfect example for exchange and flow of knowledge. It is important not only for physics, but also for its close relation with the broader scientific community, and important for contributing to individual development as well as personal relationships among scientists.

### 3.1. The Scientific Program

**Figure 6:**
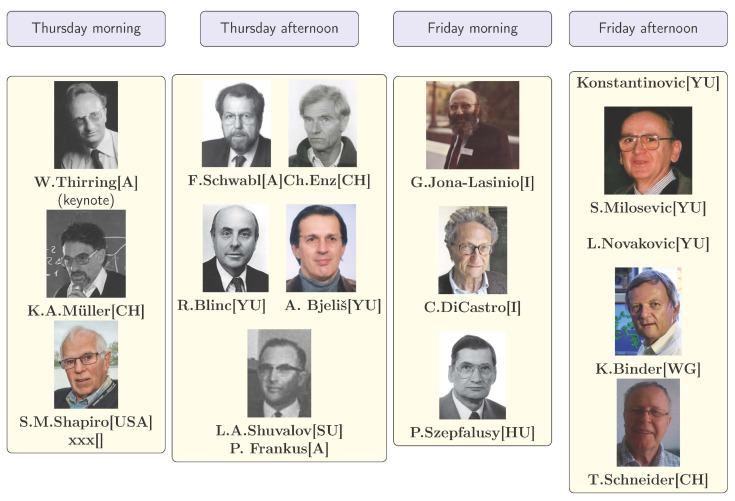
Program of the first MECO and their speakers. The name of one speaker (xxx) could not be reconstructed from the handwritten notes.

Although the author participated at the first MECO, he has only weak memories on the meeting. The notes below are based on the remarks made on the Provisional Program. Two talks have been canceled (the one of R. A. Cowley from Edinburgh UK and of S. Großmann from Marburg West Germany). Further talks have been included to give a total of 18 talks at the two days (see Figure 6). One name of a speaker (xxx) is missing since it could not reproduced from the handwritten notes.

The speakers from the eastern side of the Iron Curtain where from Yugoslavia, Hungary, and the Soviet Union. The ratio between speakers from the western side of the Iron Curtain to the eastern side was 9 to 8. The first version of the provisional program had a ratio of 5 to 1! No list of participants is available, so nothing can be said about the number of young scientists participating. Such participation lists were only available since 1977 (MECO 4).

The content of the program can be reconstructed by publications shortly before or after MECO 1. The above-mentioned Third International Meeting on Ferroelectricity is such a case. Many talks were published in the conference proceedings. According to the changed program, Thursday morning contained four talks:

- W. Thirring (Vienna) presented a keynote lecture discussed below;
- K. A. Müller (Zürich) Critical Phenomena at Structural Phase Transitions (SPTs) as Measured by Paramagnetic Resonance [53];
- S. M. Shapiro (Brookhaven) Neutron Scattering Studies of SPTs in SrTiO${}_{3}$ and KMnF${}_{3}$ [54];
- An unidentified speaker probably from an Eastern country.

On Thurday afternoon six talks were presented:

- F. Schwabl (Linz) Critical Dynamics and Microscopic Theory of the Central Peak at SPTs [55];
- Ch. Enz (Geneva) probably on the Hydrodynamic Theory of the Central Peak [56];
- R. Blinc (Ljubljana) Underdamped Soft Modes in Order Disorder Systems [57];
- A. Bjeliš (Zagreb) probably on Structural Instabilities in One-Dimensional Systems [58] the topic of his theses supervised by S. Barišić;
- L. A. Shuvalov (Moscow) probably about Raman-Scattering [59];
- P. Frankus (Vienna) probably on Galvanomagnetic Properties [60].

Friday morning contained three talks:

- G. Jona-Lasinio (Padova) An Introduction to the Renormalization Group Approch to Critical Phenomena [61];
- C. Di Castro (Rome) Renormalization, Present Status [62];
- P. Szépfalusy (Budapest) Application of Wilson’s approach to Dynamic Critical Phenomena [63].

On Friday afternoon five talks were given:

- J. Konstantinović (Belgrade) probably on antiferromagnetic phase transitions [64];
- S. Milošević (Belgrade) probably on the Ising Model [65];
- L. Novaković (Belgrade) published 1975 a book in the field of magnetic and ferroelectric phase transitions [66];
- K. Binder (Munich) Monte Carlo Experiments on Critical Phenomena and Metastable States [67];
- T. Schneider (Zürich) Molecular Dynamics Investigation of STPTs (slowing down of cluster dynamic, giving size to central peak phenomena) [68].

A remarkable feature of the meeting remained the confrontation of theoretical, numerical, and experimental results. The new field of computer simulations was not generally accepted in the physical community without reservation. It was questioned how these simulations can contribute to our understanding of the phenomena. As an example, Michael Fisher’s view might be cited: “If one had a large enough computer to solve Schrödinger’s equation and the answers came out that way, one would still have no understanding of why this was the case” [In [69] p. 46]. Later he affirmed the method: “By carefully analyzing numerical data from relatively small finite systems using scaling and extrapolation methods, it is demonstrated that one can reliably estimate critical exponents, critical temperatures, and universal amplitude ratios, thereby distinguishing convincingly between different *nearby* universality classes and revealing systematic crossover effects” (from the abstract of [70]).

### 3.2. The Vienna School of Statistical Thought

No title was given for W. Thirring’s keynote lecture, but he may have reported recent work on ‘Exact results on the structure of matter’, a theme he had presented at the Internationale Universitätswochen für Kernphysik in Schladming [71] a year ago, and which was followed by a cooperation with E. Lieb and the papers on stability of matter. At this meeting in Schladming also K. Wilson gave a talk on ‘Exact and Approximate Forms of the Renormalization Group’; however, this was not edited in the proceedings.

**Figure 7:**
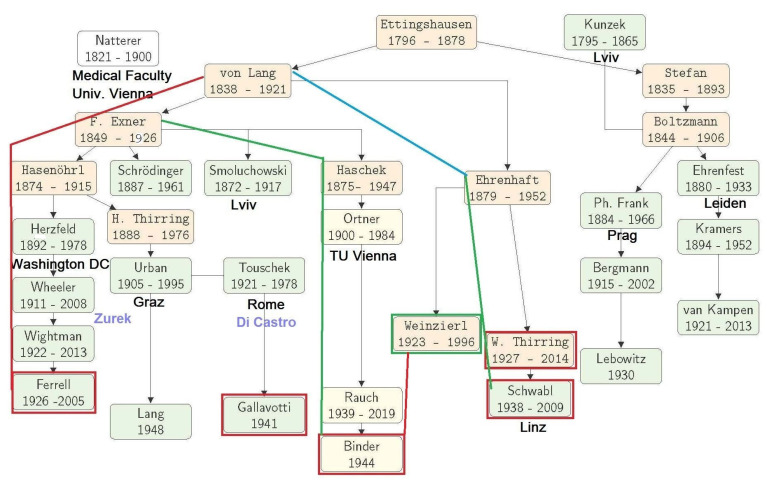
The person-to-person network (physical tree) of the ‘Vienna School of Statistical Thought’. The lines with arrows indicate the supervisor–student relation; lines without arrows indicate a teacher–student relation. Color of the box: green came to or left the University Vienna to the places indicated; red frames theoreticians, green frames experimentalist related to MECO. For further details see text.

Thirring may have also pointed to the tradition the research in statistical physics at the Physical Department of the University of Vienna. It was Elliot Montroll ten years later 1984 who described this tradition by defining and explaining the ‘Vienna school of Statistical Thought’ [72]. He explained the flow of knowledge in the academic genealogy of thesis advisors [73], known as physics tree (in our field). A modified version of this tree adapted to this paper and the history of critical phenomena is shown in Figure 7 (see also [74])

There are two branches (red and blue lines in Figure 7) of the tree connecting Richard Ferrell and Franz Schwabl. They end in a bifurcation almost a century ago at Victor von Lang with the arms represented by Franz Exner and Felix Ehrenhaft. This branch reached USA when Karl Ferdinand Herzfeld emigrated in 1926 on a guest-professorship at the John Hopkins University at Baltimore in Maryland. In fact, also K. Binder has such a connection (green line in Figure 7) to R. Ferrell via the branch point F. Exner.

There is also a relation to the Italian group, not as an arm of the tree but by Bruno Touschek who started his physics studies in Vienna. After the Annexion of Austria in 1938, because of his Jewish mother he had to leave the university in 1941 and went to Germany and lived undercover in the flat of W. Lenz in Hamburg (for his life see [75,76])! After his rescue, Touschek arrived in Italy where he became the father of the Italian electron collider. Di Castro remembers: “I became interested in statistical mechanics and in the theoretical physics of condensed matter, largely ignored as research topics in Rome, at a time—end of the 1950s—when everyone was engaged in the study of elementary particle physics. The course in statistical mechanics was given by Bruno Touschek, who was brilliant and thus certainly stimulating. Statistical mechanics was not his field, however. His course was based on the short book Statistical Thermodynamics [1957] by E. Schrödinger.” Touschek was also the supervisor of Gallavotti’s thesis.

The tree shows also a connection to the experimental group of Peter Weinzierl. He held a seminar on liquids and was also interested in critical phenomena. “Kurt Binder had been attending a seminar on the Physics of Liquids, organized by Peter Weinzierl (red line in Figure 7), there he became aware of the MC method and its possibility to calculate correlation functions directly and without approximation” [77]. This seminar was still running when Schwabl came to the physics institute, and the author worked on his thesis.

Results of the Weinzierl group were presented in the subsequent MECO meetings. Weinzierl chaired a session at MECO 2 where W. Grabner presented Rayleigh scattering results at the phase transitions in liquids, and K. Hanisch reported at MECO 3 and MECO 5 Mößbauer measurements at the structural phase transition in KMnF${}_{3}$ [78].

Another experimental group consisting of Frankus, Kuchar, and Valenta studied (among other things) ferroelectrics. It was in the year 1912 when Erwin Schrödinger submitted his ‘Habilitations-Schrift’ at the University of Vienna and published a ‘Vorläufige Mitteilung’ that he coined the term ‘ferroelektrisch’ (ferroelectric) [79,80]. He speculated that based on Debye’s model in solids this phase is reached like in magnets at low enough temperature. A new research group was established in 1972 around Hans Warhanek when he became professor at the 1. Physical Institute of the University of Vienna. His group continued research in the field of structural phase transitions and ferroelectrics, and Warhanek joined the advisory board of MECO.

### 3.3. Conferences in Budapest Shortly after the First MECO

The 2nd EPS Conference of the Condensed Matter Division on Dielectrics and Phonons was held in Budapest, 21–25 October 1974. “Twenty four countries were represented by 408 participants: 234 came from the Western countries, 63 were Hungarian, and the rest from other Socialist countries. Twenty one invited papers and 194 contributed papers were presented, generally in three parallel sessions” [81]. In the session on phase transitions, structural phase transitions were of greatest interest. Therefore, it was not surprising that participants of the first MECO also presented their results.

Szépfalusy organized in the following year the IUPAP (International Union of Pure and Applied Physics) Conference on Statistical Physics in Budapest [82,83]. It was the first conference to award the Boltzmann Medal, and the recipient was Kenneth G. Wilson. There were 11 invited talks and 199 contributed papers. The conference was attended by 342 scientists from 29 countries. Under the invited talks were also the MECO 1 speakers K. Binder and G. Jona-Lasinio. Two invited speakers were from Eastern countries: Ja. G. Sinai and A. A. Abrikosov.

The IUPAP was already founded in 1922 and the Commission on Statistical Physics after the Second World War in 1945. The first meeting in Statistical Physics was in Florence (Italy) from 17 to 20 May 1949. IUPAP “has had a special concern for the free movement of scientists from one country to another…[it] has struggled, for example, to insist that no physicist be barred for political reasons from an international conference organized by one of its Commissions. This is usually done by means of refusing visas” [84]. This principle of freedom for scientists was reaffirmed several times at last by the General Assembly held in Munich in 1975.

## 4. The Development of MECO

The evolution of scientific interest in physics was studied recently [85]. The authors concluded

(1) Condensed Matter is the starting field of many physicists, that then move to Interdisciplinary, Classical, and General Physics.
(2) And they found that although the majority of physicists change the topics of their research, they stay within the same broader area, thus exploring with caution new scientific endeavors.

This of course, apart from the political changes, happened also for the development of MECO. It started from sharing a rather well-defined problem from which it expanded more and more in other fields. This, on one hand, was due to the underlying common physical concepts and, on the other hand, due to personal careers and new cooperation of the participating scientists. Here this expansion is only sketched in its simplest elements.

### 4.1. MECO until the Yugoslavian War

The organization of MECO 1 established a form of a new type of conference similar to a combination of the European Study Conferences and Schools of the EPS (see above Section 2.3). On one hand a better balance between East and West was intended, on the other hand the meeting should be open to aspiring scientists. The organization of the MECO Meetings also runs along these lines. In particular:

- Each year changing the side of the Iron Curtain for the meeting place.
- All participants accommodated in one location (this sets a limit to about 100 participants), first realized in 1976, at MECO 3 and present (with exceptions) for the whole time of the meeting (three days).
- No parallel sessions as usually at large conferences.
- Free stay for at least participants from the eastern side, eventually support of travel expenses. For western participants a small amount covered the stay (e.g., for the stay in Liblice castle (MECO 13) 50 U.S. dollars had to be paid).
- Participants mainly from *MECO centers* but in principal open to all scientists.
- No proceedings (with rare exceptions MECO 19 and MECO 45). The idea was to report on ongoing research, which contained recently published material and intended publications.
- Invited review talks (chosen by the organizers in cooperation with the advisory board), contributed talks (selected out of applications for a talk otherwise shifted to the poster session), and posters (since 1977 MECO 4).
- Reports from different groups about research plans, topics of future seminars, etc.

**Figure 8:**
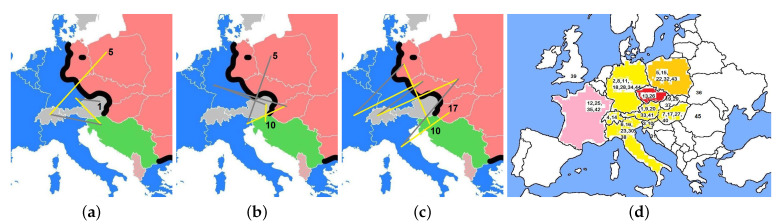
MECO meetings from (**a**) the first (1974) to the fifth (**b**) from the fifth to the tenth and, (**c**) from the tenth to the seventeenth on the map of Europe during the cold war (blue NATO states, red Warsaw pact, light red Albania, green Yugoslavia, thick black line Iron Curtain). The yellow line indicates crossing the Iron Curtain from west to east. (**d**) All MECO meetings (subscripted by their number) on the map of the present state of Europe. For further details see text, and for a more precise location of the meetings see the Wikipedia page Middle European Cooperation in Statistical Physics.

Contrary to the aim of crossing the Iron Curtain the MECO 2 took place in West Germany. It may be that the organization in Hungary, which might have been the natural next place, was not possible because Szepfalusy was organizing the IUPAP conference. With U. Krey, W. Gebhardt, G. Obermair, W. Dultz, and J. Keller, a group of physicists was working in the field of phase transitions and critical phenomena at the new University of Regensburg. Only some years later the intended zig-zag change of meeting places through the Iron Curtain could be reached (see the maps in Figure 8).

It also took some time to find a suitable structure of presenting the results of the participating scientists. Poster sessions first appeared in the late 1960s, but it got the appropriate acceptance at the American Physics Conference 1975 [86]. At MECO meetings such sessions were introduced in 1977 at MECO 4. It was an important step to increase the exchange of ongoing work in the different groups and opened the possibility (especially for young scientists) to present very recent—so far unpublished-work. The poster were accessible during the whole time of the meeting.

At the MECO 4 meeting in Switzerland for the first time East Germany participated, and the researchers presented their results. It was the group around Adolf Kühnel from University of Leipzig. “In the ‘Solid State Theory’ working group founded in 1972 in the course of the third university reform in the physics section, Kühnel soon dealt with the theory of phase transitions, with Steffen Trimper and Thomas Nattermann in particular. The study of the theory of structural phase transitions in ferroelectrics took place partly in collaboration with experimental physicists in Leipzig, in particular from the working group ‘Physics of Condensed Matter’ by Artur Lösche, and in Halle with Horst Beige” (from [87], translation by the author).

The first advisory board was named at first in the second circular of MECO 5. It consisted of R. Blinc, G. Meissner, K. A. Müller, P. Szepfalusy, and H. Thomas. Later in the program it was enlarged as can be seen in Table 1.

Then, step-by-step more interested groups organized MECO meetings behind and before the Iron Curtain: Poland (MECO 5), East Germany (MECO 11), France (MECO 12), and Czechoslovakia (MECO 13). This also increased the international advisory board, which was named officially in the program of MECO 5. Usually at a MECO meeting the places for the next two years were decided, and if the possible organizers agreed the decision was announced. From these new countries group leaders were included into the advisory board. This led to a continuous increase in the advisory board (see Table 1).

At MECO 18 (in Duisburg, Germany 12–14 March 1991) the advisory board envisaged MECO 19 to take place from 31 March to 2 April 1992 in Beograd and MECO 20 in Greece. However just fourteen days later on 31 March 1991 the Yugoslavian war began and lasted ten years. As a result, a breakup of Yugoslavia began and lasted several years. It started in 1991 with Slovenia and Croatia and ended now in total in six states and Bosnia and Herzegovina, Serbia, Montenegro, North Macedonia, and Kosovo, whose status is disputed (for further details see [88]). Thus, the next MECO meeting could not take place, and the future of MECO remained unclear. In the meantime, however, the Iron Curtain was torn down, the German reunification occurred, and a partial fragmentation of the Soviet union took place (see the map in Figure 8c).

### 4.2. Restarting MECO

In the year 1993 at the German Physical Society meeting in Regensburg, F. Schwabl and P. Szépfalusy asked A. Surda from Slovakia and the author if they could organize a meeting (MECO 19) in the next year. This was possible for Surda, and the meeting started with 18 invited talks 55 posters. Since this restart was a special event, proceedings of this meeting [89] were published. The author had the possibility to organize then in 1995 MECO 20 in Austria. Thus, a restart of the MECO was made possible, and the meeting has taken place every year since.

**Figure 9:**
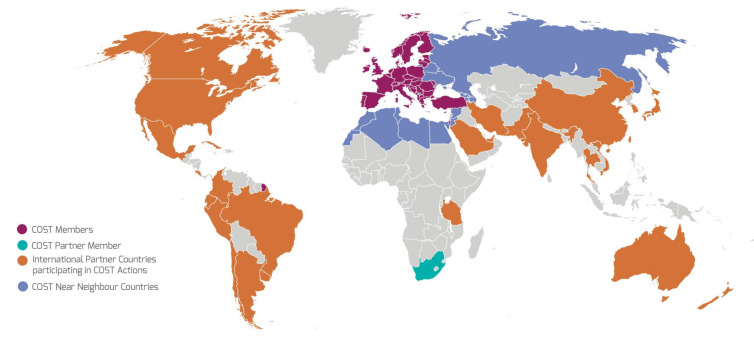
COST actions and participating countries; all countries in Europe participating until 1991 that now belong to COST countries (red) (from [90]).

However, the general circumstances have changed a lot. Most important were the changes in the political situation in Europe: the fall of the Iron Curtain, the changes in the enlargement of the European Community, and the economical changes for the scientific community by the development of European research programs. Two programs should be named: INTAS for grants to support research cooperation with the New Independent States (founded 29 June 1993) and COST (see Figure 9). This action was dedicated to scientific collaboration and complementing national research funds. It was in fact founded already in 1971 and is the longest-running European framework for research and innovation. Researchers affiliated with institutions in Near Neighbor Countries can participate in COST Actions on the basis of ascertained mutual benefit subject to approval.

The strategy of the action highlighted “the importance of interdisciplinary bottom-up networks as impactful tools to bridge the participation gap and close the innovation divide in Europe while providing opportunities for younger generations.” (Preamble in [90]) This is pretty similar to the MECO concept, although it was never written down.

The increase in the number of countries belonging to the European Community and the possibility to access financial support for research programs made it necessary for the organizers of MECO to introduce conference fees. Each organizer had—as before 1991—to care for the financial support, the gain was the remaining independence from restrictions made by larger organization and flexibility. The fee usually covered accommodation, meals, and social events. However, it remained possible to support participants from non-EU countries and students. Moreover, national institutions also supported the cooperation with non-EU countries, and this also made it possible for scientists of non-EU countries to participate at MECO conferences.

Another important step in sharing knowledge was the foundation of ArXiv—the preprint server—by Paul Ginsparg, a Ph. D. student of Kenneth G. Wilson in the year 1991 [91]. One should remember that at the beginning of the 1970s there were places where “at that time there was no physics colloquium with any speakers from abroad, …there has not yet existed the internet at the time, international phone calls were discouraged as too expensive, and…personal contacts to established scientists were restricted to sending them postcards requesting reprints (and possibly preprints)” [17].

Without going into detail in this publication, the interdisciplinary character of contributions also increased at MECO meetings. At MECO 37 (2012) under the title ‘Interdisciplinary applications’ contributions were announced for the first time, but in the program they were not identified. Only at MECO 42 (2017) could contributions under this title be identified.

The spreading of ‘Statistical Thoughts’ can be described as in a Report to the 2002 General Assembly of the IUPAP commission for Statistical Physics by the chairmen Kurt Binder [92]. There under the title ‘New Developments’ it was mentioned that “research in statistical physics maintains an interdisciplinary character, both within physics and even outside of it”. This also holds for the development of the MECO meetings. In order to give a specific example one can list the following selection of talks:

MECO 4 (1977): K. Binder Phase transitions in systems with disorder
MECO 6 (1979): C. De Dominicis Systems with Quenched Random Impurities Including Spin Glasses
MECO 16 (1989): I. Kondor Fluctuations and chaos in the Ising spin glasses
MECO 25 (2000): I. Kondor Spin glass effects in finance
MECO 43 (2018): J.-P. Bouchaud Statistical physics in economics and finance

“Econophysics originated in the 1990s, simultaneously in two different places. Eugene Stanley in Boston, USA, and Imre Kondor in Budapest, Hungary, as professors of physics allowed their students to write their graduate theses on statistical physics by applying financial data.” [93]. It demonstrates what was formulated by D. Sherrington (a speaker of MECO 20): “The scientific journey of discovery has been highly interdisciplinary, and there is much more scope, in many directions.” [94]. Even earlier it was noted: “We illustrate the general principle that in biophysics, econophysics and possibly even city growth, the conceptual framework provided by ‘scaling and universality’ may be of use in making sense of complex statistical data” (from the abstract of [95] accentuation by the author). Indeed, in 2006 at MECO 31, S. Galam gave an invited lecture on sociophysics (for a short introduction see, e.g., [96]).

On the other hand, the tight connection to experimental research was weakened over the time. To quantify this change, deeper analysis of the contributions to MECO is necessary. At least at the meeting of the advisory board at MECO 33 this observation was on the agenda.

The development of MECO geographically and personally together with the scientific changes which were identified with the term ‘Statistical Thought’ led to a new orientation of the content presented at MECO meetings. This has been expressed by the organizers of MECO 43: “ Today MECO attracts scientists working in the field of statistical physics and related areas from the whole Europe and other continents. It covers the whole spectrum of topics ranging from ‘statistical mechanics, condensed matter, soft matter, active matter, quantum many body systems and quantum information, frustrated and disordered systems, complex systems, complex networks, classical and quantum critical phenomena, non-equilibrium phenomena’ to interdisciplinary applications of statistical physics and it follows current developments in these fields and related areas. …This year there are many contributions on interdisciplinary applications of statistical physics including those in ‘finance, social sciences, complex system, biophysics and information theory’.” (accentuation by the authors).

Thus, MECO as a bottom-up undertaking of at least two generations of scientists showed enough flexibility concerning its scientific content and enough engagement concerning its organization to survive the political, economic, and scientific changes happening in the 1990s in Europe.

### 4.3. The Development of MECO in Simple Numbers

**Figure 10:**
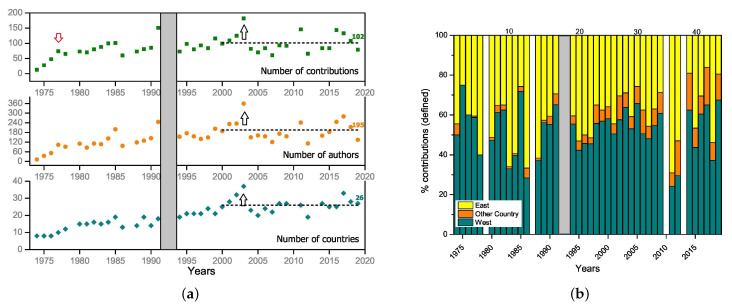
(**a**) Number of presentations, authors, and participating countries; (**b**) distribution of the affiliation of the authors’ institution in the contributions (invited talks, contributed talks, posters) western or eastern of the Iron Curtain or in other countries. The upper numbers counts the MECO meetings. For further details see text.

The data material for an analysis consists of the available programs of the MECO meetings. There one can find the different contributions, the names of the authors with their affiliation, the title, and abstracts. In addition, one has the list of participants of the meeting, the local organizing committee, and the advisory board. A glimpse of a quantitative analysis of this data is given by the two plots in Figure 10. Figure 10a shows the stabilization of the numbers of contributions, authors, and countries contributing to the meetings.

The increase in contributions goes along with the increase in participating countries in the first years, and since MECO 4 1977 (see left arrow in Figure 10a) it is due to the poster session. Concerning the number of authors and their distribution over the presentations is dependent on the presentation of the program by the organizers, in some cases only the speaker or the presenting person at the poster is denoted in the program.

The peaks in the number of contributions are related to the place where the MECO meeting takes place. If it is in a city with universities or nearby there are additional contributions from authors, which participated only on a day-by-day basis. This is seen 1991 in Duisburg, 2003 in Saarbrücken, 2011 in Lviv, and 2016 in Vienna. One of the largest meetings was MECO 28 from 20 to 22 March 2003 on the campus of the Saar University (see right arrows in Figure 10a). About 180 scientists from Europe met to keep up to date with current developments in the field of Condensed Matter Physics and Statistical Physics. More than one-third of scientists from former Eastern countries participated.

The percentage of Western and Eastern contributions at the MECO meetings is shown in Figure 10b. The country of the affiliation of the authors contributing is taken for the classification Western, Eastern and Other Countries (non European). After 1991 former East German institutions are counted as Western countries, all other new Eastern countries remained Eastern. The gaps (MECO 6, 14, 35, 38) are due to insufficient data (e.g., incomplete list of contributions are available and/or not all authors are shown). As one would have expected, especially for the period before 1991, the Eastern contributions are largest when the MECO takes place in an Eastern country.

A more specific preliminary analysis has been presented at MECO 45 by Olesya Mryglod [97] and will be published within the project [8]. It contains, e.g., a more detailed geographical, authorship, and collaboration analysis.

## 5. Discussion

The flow of knowledge depends on the possibilities of the scientists to create connections between each other. Looking at the history of MECO, the creation and development of these possibilities can be seen. Several important developments outside the scientific community have been named. There is the willingness for bottom-up self-organization, the change of the political situation (breakdown of the Iron Curtain, expansion of the academic network), funding by local and international institutions of movements of scientists, and the development of new communication possibilities (internet, publishing facilities). The goal of all of this is to enable easy personal and face-to-face contacts.

There are many reasons hindering these contacts such as wars, economical crises, or as it is now at MECO 45 a pandemic. Fortunately, technical devices now allow meetings such as MECO via internet, but essential features which had the founders of MECO in mind cannot be realized. The component of the social contacts is missing. On the other hand, the experience with the internet at conferences, in teaching, and researching at the universities might become of more importance in the future even if the pandemic restrictions are abolished. A combination of personal attendance and internet participation could improve the sharing of knowledge. It would also allow documentation of research results similar to preprints, proceedings, and publications. It also could support the flow of knowledge into different disciplines.

An active scientific network may be considered as a network of agents that are in constant competition and collaboration. Therefore, it holds as Imre Kondor in 2019 at the Complexity Science Hub stated [98]: “Such a system is never in equilibrium. Rather it executes movements, shifts, fluctuations around a more or less well-defined working point. This state is flexible, can accomodate to changes in the environment without loosing its identity. It might happen, however, that a major crisis totally upsets this delicate system of collaboration and competition, excitation and inhibition, check and balances”. So far MECO has mastered all these crises, and the author expects this to hold for the future.

## Figures and Tables

**Table 1:** Examples of the Advisory Board of MECO meetings showing its increase until the interruption by the Yugoslavian War. In the last line the ratio between members from Western to Eastern European countries is given. The country index is defined by the place of institution where the scientist was employed.

MECO 5 1978	MECO 8 1981	MECO 9 1982	MECO 18	1991
R. Blinc (YU)	R. Blinc (YU)	D. Bäuerle (A)	D. Bäuerle (A)	S. Milošević (YU)
C. Di Castro (I)	C. Di Castro (I)	K. Binder (WG)	K. Binder (WG)	K. A. Müller CH)
C. Enz (CH)	V. Dvorak (CZ)	R. Blinc (Y)	R. Blinc (Y)	R. Pick (F)
G. Meissner (WG)	C. Enz (CH)	C. Di Castro (I)	M. Capizzi (I)	L. Picman (YU)
K. A. Müller (CH)	Z. Galasiewicz (PL)	V. Dvorák (CZ)	E. Courtens (CH)	A. Rigamonti (I)
L. Picman (YU)	A. Kühnel (EG)	C. Enz (CH)	C. Di Castro (I)	T. Schneider (CH)
P. Szépfalusy (H)	K. A. Müller (CH)	Z. Galasiewicz (PL)	V. Dvorák (CS)	F. Schwabl (WG)
H. Thomas (CH)	L. Picman (Y)	A. Kühnel (EG)	Ch. Enz (CH)	P. Szépfalusy (HU)
H. Warhanek (A)	A. Rigamonti (I)	G. Meissner (WG)	Z. Galasiewicz (P)	J. Snajd (P)
	F. Schwabl (A)	K. A. Müller (CH)	W. Kleemann (WG)	H. Thomas (CH)
	P. Szépfalusy (H)	L. Picman (Y)	I. Kondor (H)	V. Tognetti (I)
	H. Thomas (CH)	A. Rigamonti (I)	A. Kühnel (EG)	J. C. Toledano (F)
	H. Warhanek (A)	P. Szépfalusy (H)	G. Meissner (WG)	S. Trimper (EG)
		F. Schwabl (WG)	F. Mezei (WG)	J. Villain (F)
		H. Thomas (CH)	F. Milia (GR)	H. Warhanek (A)
		H. Warhanek (A)		
West/East ∼ 2:1	West/East ∼ 7:6	West/East ∼ 5:3		West/East ∼ 2:1

## Data Availability

Data supporting reported results can be found on the web page https://sites.google.com/site/mecoconferencephysics/products-services.
